# The Psychological Impact of COVID-19 Among Pakistani Adults in Lahore

**DOI:** 10.3389/fpubh.2021.578366

**Published:** 2021-04-21

**Authors:** Saima Majeed, Elizabeth Maria Schwaiger, Abia Nazim, Ivan Suneel Samuel

**Affiliations:** ^1^Department of Clinical Psychology, Punjab Institute of Mental Health, Lahore, Pakistan; ^2^Department of Psychology, Forman Christian College: A Chartered University, Lahore, Pakistan

**Keywords:** psychological impacts, COVID-19, corona virus anxiety, obsessions related to COVID 19, Pakistan

## Abstract

**Background:** In the wake of the worldwide spread of the novel coronavirus and the resultant restrictive measures, mental health has become a crucial issue. Physical health is not the only aspect of humans that is at risk. Globally, the rates and severity of mental illness are being significantly impacted by this pandemic. Two scales have been validated to measure the impact of coronavirus disease 2019 (COVID-19) on the levels of anxiety and obsessional thinking in clinical and non-clinical populations. The present study was designed to investigate the levels of anxiety and obsessions related to COVID-19 in the general public of Lahore, Pakistan.

**Materials and Methods:** Data were collected *via* snowball sampling from May 9 to May 19. An online survey consisting of a demographic profile and two scales, Coronavirus Anxiety Scale (CAS) and Obsession with COVID-19 Scale (OCS), was sent through email, WhatsApp, and Facebook groups to adults (18 years and above) of Lahore, Pakistan.

**Results:** A total of 240 individuals (20% men and 80% women) recorded their responses. The majority belonged to a nuclear family system (60%), and their education level ranged from high school to Ph.D. The cut-off score for probable dysfunctional coronavirus anxiety and obsession levels was not met within this sample (CAS, *M* = 3.24, *SD* = 4.21; OCS, *M* = 4.14, *SD* = 3.15), suggesting that the general population of Lahore, Pakistan is not suffering from dysfunctional anxiety or obsessions related to COVID-19. Forty-seven participants' score on OCS and 35 participants' scores on CAS were above the cut-off, i.e., ≥7 and ≥9, respectively. The results of the correlation analysis showed a significant positive relationship (^**^p < 0.619) between anxiety and obsessions related to COVID-19.

**Conclusion:** One important, yet surprising, conclusion of this study is that the average adult in Lahore does not show much anxiety or obsessions related to COVID-19. Other studies around the world using these measurement tools have indicated significantly high levels of both anxiety and obsessions related to COVID-19. These findings may demonstrate the resilience of Pakistanis or perhaps the lack of understanding of the seriousness of the situation.

## Introduction

Viral epidemics continue to emerge and pose serious threats to public health. Even in the past 20 years, the breakdown of many viral epidemics including severe acute respiratory syndrome coronavirus (SARS-CoV) in 2002 and 2003, H1N1 influenza in 2009, and Middle East respiratory syndrome coronavirus (MERS-CoV) in 2012 has been recorded (1). The outbreak of coronavirus disease 2019 (COVID-19) in China in December 2019 has rapidly proliferated in more than 200 countries around the globe, not only paralyzing daily lives but also creating insurmountable challenges for mankind (2). Historically, the large-scale epidemics and pandemics had generally left long-standing social and psychological impacts on mankind (3). The novel coronavirus (COVID-19) pandemic has not only emerged as a major health threat throughout the world but also brought many social, financial (4), and psychological (5) challenges with it (2). This large-scale pandemic has adversely impacted the lives of a significantly large population of the world (6), particularly leaving more pressure on those living in underdeveloped and developing countries, such as Pakistan.

Pakistan reported its first case of coronavirus in February 2020 and implemented a nationwide lockdown in the third week of March 2020 to contain the situation by flattening the curve of the spread of the disease. In compliance to lockdown measures, all educational institutes, all significant public places including shopping malls, religious worship places, and restaurants, and many industries were closed. The lockdown led to many problems that were likely to increase the psychological burden of the population even in developed countries (3).

Pakistani society significantly values socialization and social activities, and people give lots of importance to social interaction. The implementation of lockdown and social distancing created disruption in many routine activities, consequently increasing the emotional pressure on people. Financial constraints and pressures also contributed to the psychological difficulties as a large segment of the Pakistani population works in the private sector or belongs to the daily wage workforce with 24% of the total population reported to be living below the poverty line and 38.8% of the population reported to be poor (7). As a result of the complete lockdown, the country has faced a serious halt to economic activities (8); many workers have either lost their jobs or faced significant cut in their salaries, contributing more to the already existing financial difficulties, resulting in increased cases of suicide (9). Since the emergence of COVID-19, many conspiracy theories and myths about the condition have been spreading in Pakistan, which also resulted in developing fears and anxiety among the general public (10). Moreover, these lead to more uncertainty and increased unrest among the public. All these factors stress the need to explore the psychological influences of the coronavirus pandemic in the general population.

There are many researchers studying different medical (11), social, and psychological influences (12) of coronavirus in different countries, and they reported contradictory findings. In China, Tian et al. (13) concluded that more than 70% of their sample had moderate to severe levels of different psychological symptoms. They also observed that females and younger participants had higher levels of psychological disturbance. Varshney et al. (14) studied the psychological effects of coronavirus in the general population of India. They reported that one-third of their sample was psychologically affected by the coronavirus pandemic and females and younger participants and those with an existing physical illness were significantly more affected than others. Salman et al. (10) studied the impact of coronavirus in Pakistani university students and observed that 34% of their sample had moderate to severe levels of anxiety and 45% had moderate to severe levels of depression. Female respondents and those below 30 years old were more depressed, whereas participants with a friend or relative diagnosed with coronavirus reported more anxiety. In light of the literature review, the present study was designed to explore the psychological impact of the COVID-19 pandemic on the Pakistani population. We hope that the findings will contribute to understand the requirements of the population and to design effective strategies to help people cope with the psychological burden of the current pandemic.

## Materials and Methods

The present study followed the survey method for data collection. Through convenient and snowball sampling technique, data were collected from the adult population (18 years and above) of Lahore, Pakistan from May 9 to May 19, 2020. A Google form was prepared and sent through email, WhatsApp, and Facebook groups to the contact list of the authors. Then, these groups were further requested to send the form into their own contact list. The snowball sampling technique was used to get a quicker sample that allowed the study to take place at the perfect time; with the restrictions placed during the lockdown, it was the best way to reach participants. This method was cost effective and simple as data were collected by the primary source and the research had no external funding. Moreover, this sampling technique needed less planning and workforce than other sampling techniques. The online survey was composed of the demographic information sheet, Coronavirus Anxiety Scale [CAS; (15)], and Obsession with COVID-19 Scale [OCS; (16)]. CAS is a 5-item scale, rated across a 5-point scale from 0 (not at all) to 4 (nearly every day). It measures an individual's experience of anxiety related to coronavirus during the past 2 weeks. The score range is 0–20, and higher scores indicate higher anxiety. The cut-off score has been established, and scores equal to or above 9 are considered to be problematic and the individual has to be referred for further assessment and treatment (15). The author reports that it is a reliable and valid scale with a Cronbach's alpha value of 0.93. The scale also has high diagnostic properties, with 90% sensitivity and 85% specificity, when compared with other instruments, such as Generalized Anxiety Disorder-7. OCS is a self-report 4-item scale in which each item is rated across a 5-point scale ranging from 0 (not at all) to 4 (nearly every day). The scale measures an individual's experience of persistent and disturbed thinking related to COVID-19 over the past 2 weeks. The score range is 0–16, and higher scores indicate higher rate of obsessive thinking. A score equal to or above 7 indicates a problematic and dysfunctional thinking, and the individual needs to be referred for further assessment or treatment. It is a reliable (αs >0.83) and valid instrument. The author reports high diagnostic properties (81–93% sensitivity and 73–76% specificity) of the scale with related instruments, such as General Health Questionnaire (GHQ). According to Lee (16), researchers are using the CAS and OCS internationally across various cultures to assess both anxiety about COVID-19 and obsessional thinking about COVID-19.

In the present study, general information regarding the survey and process of data collection was conveyed to the participants. The inclusion criteria were that participants were above the age of 18 and could read English. The exclusion criteria were that the participants had no history of any diagnosed psychological illness and were not on any related medications. This allowed the researchers to rule out the confounding factor of the mental health of the participants. Participants were also told that their participation was voluntary and without any monetary gains. The confidentiality of their information was also an important priority of the researchers. Data were collected without their names mentioned or asked, and file was also encrypted and could be seen or used for research purpose only.

### Statistical Analysis

Both descriptive and inferential statistics were executed with the help of Statistical Package for Social Sciences version 21 (SPSS). Frequencies and percentages were calculated to determine the demographic characteristics of the sample as well as the response categories of anxiety and obsessions related to COVID-19. Pearson Product Moment Correlation analysis was also carried out to examine the relationship between anxiety and obsessions related to COVID-19. Independent samples *t*-tests were computed to compare men and women on the levels of anxiety and obsessions related to COVID-19.

## Results

Two hundred forty adults participated in the survey. Among them, 80% women and 20% men recorded their responses during the said period. Most of them belonged to a nuclear family system (60%). The range of their educational level was high school to PhD, but most of them were undergraduates and above (96%). The age range was 18–70 years (*M* = 28.15, *SD* = 8.85). Among them, 47.5% were students, 23% were working in the private sector, 16.7% were employed in the public sector, and 7.1% were self-employed. Two housewives and 11 unemployed individuals also participated in the survey, whereas only 1 participant was retired. Participants were South Asian, specifically from the Punjab region of Pakistan. They did not have any specific race as Pakistan is largely mono-ethnic and not many foreigners reside here.

The results in Table 1 showed that the mean scores of both anxiety and obsessions scales were below the cut-off. Specifically, 35 (14.6%) participants reached the cut-off for clinically significant levels of anxiety, and 47 (19.6%) reported obsessional thoughts about COVID-19 that were above the cut-off.

**Table 1:** Mean, standard deviation, and minimum and maximum ranges along with the Cronbach's alpha of CAS and OCS (*N* = 240).

**Variables**	***k***	***M***	***SD***	**Cut-off**		**Range**	**a**
					**Minimum**	**Maximum**	
CAS	5	3.24	4.21	≥9	0	17	0.90
OCS	4	4.14	3.15	≥7	0	13	0.75

*k, no of items; M, mean; SD, standard deviation; a, Cronbach's alpha*.

The results in Table 2 illustrated that approximately half of the population opted for the not at all response category and the rest of them chose other severity levels of anxiety responses ranging from mild to severe.

**Table 2:** Frequencies and percentages of responses for Coronavirus Anxiety Scale (*N* = 240).

**Variables/responses**	**CAS 1 *f* (%)**	**CAS 2 *f* (%)**	**CAS 3 *f* (%)**	**CAS 4 *f* (%)**	**CAS 5 *f* (%)**
Not at all	121 (50.4)	162 (67.5)	151 (62.9)	152 (63.3)	171 (71.3)
Rarely/less than a week	54 (22.5)	23 (9.6)	50 (20.8)	52 (21.7)	33 (13.8)
Mild/several days	30 (12.5)	23 (9.6)	21 (8.8)	21 (8.8)	21 (8.8)
Moderate/half of the days	25 (10.4)	17 (7.1)	16 (6.7)	14 (5.8)	12 (5.0)
Severe/nearly every day	10 (4.2)	1 (0.4)	2 (0.8)	1 (0.4)	2 (1.3)

The results in Table 3 indicated that more than half of the participants marked the more severe options for obsessions related to COVID-19.

**Table 3:** Frequencies and percentages of responses for Obsession with COVID-19 Scale (*N* = 240).

**Variables/responses**	**OCS 1 *f* (%)**	**OCS 2 *f* (%)**	**OCS 3 *f* (%)**	**OCS 4 *f* (%)**
Not at all	79 (32.9)	69 (28.2)	67 (27.9)	181 (75.4)
Rarely/less than a week	75 (31.3)	87 (36.3)	74 (30.8)	30 (12.5)
Mild/several days	63 (26.3)	55 (22.9)	57 (23.8)	14 (5.8)
Moderate/half of the days	20 (8.3)	22 (9.2)	28 (11.7)	10 (4.2)
Severe/nearly every day	3 (1.3)	7 (2.9)	14 (5.8)	5 (2.1)

The results in Table 4 showed a significant positive relationship between anxiety and obsessions related to COVID-19.

**Table 4:** Relationship between anxiety and obsessions related to COVID-19.

**Variables**	**Anxiety**	**Obsessions**
Anxiety		0.617^**^
Obsessions		

** *p > 0.01*.

The results in Table 5 depicted significant gender differences regarding both anxiety and obsessions related to COVID-19. Men scored higher on both the scales than women.

**Table 5:** Independent samples *t*-test for gender differences for anxiety and obsessions related to COVID-19 (*N* = 240).

**Variables**	**Men (*****n*** **=** **49)**		**Women (*****n*** **=** **191)**		***t*_**(238)**_**	***p***	**95% CI**	
	**M**	**SD**	**M**	**SD**			**UL**	**LL**
CAS	4.59	5.03	2.89	3.91	2.54	0.01^**^	0.38182	3.01128
OCS	5.18	3.69	3.88	2.69	2.58	0.01^**^	0.31034	2.29503

** *p < 0.01; M, mean; SD, standard deviation; CI, confidence interval; LL, lower limit; UL, upper limit*.

## Discussion

All over the world, rates of mental illness–particularly related to coronavirus–have increased since the advent of the pandemic. Other studies have found a significant psychological impact of the coronavirus in India (17) and Pakistan (10); however, in this sample, the average level of anxiety and obsessional thoughts due to COVID-19 is below the cut-off scores. A large majority of the sample reported little to no anxiety or obsessions about coronavirus, with 14.6% reporting clinically significant dysfunctional anxiety about coronavirus and 19.6% reporting problematic obsessions about COVID-19. This is in contrast to rates of depression, anxiety, and stress in Pakistani undergraduate students (10). Possible explanations for this discrepancy could be multifactorial. The study by Salman et al. (10) was conducted on undergraduate students, and one of the findings of the study was that age was a factor in the levels of mental illness. Those above 31 years old had lower levels of depression than those below 30 years old. The current study's sample had an average age of 28.15 years, thus presenting a possible reason for lower rates of reported distress. Additionally, the current study did not evaluate the diagnosis of COVID-19 among family members, which is a strong correlation of anxiety.

There are several other possible explanations for this low level of clinical distress on average among this group. When this study was conducted, the lockdown was in effect, and the number of cases was still quite low. The study by Salman et al. (10) was conducted prior to the lockdown, when there was greater uncertainty. Moreover, it is possible now that the cases are rising rapidly, the full psychological impact of the COVID-19 pandemic will be more apparent. Further research should be conducted to continue to monitor the levels of psychological distress and ascertain the needs of the country.

Another possible explanation is that Pakistan is a developing nation, where diseases, such as malaria and dengue fever, are seasonal realities, where polio is still not eradicated, and where massive political upheaval is historically recent. Conceivably, in such an environment, COVID-19 is just another change to adjust to or risk to manage as people go about their day-to-day lives. Pakistan is also a collectivistic society. In such societies, reporting of mental illness is often lower, due to many factors (17). Perhaps, these rates of psychological distress are in fact higher than reported or expressed in a different way. Future research should consider this factor.

Several studies have indicated that the two measures used in this study are correlated with general distress (15). The present study also found a strong correlation between the two measures, suggesting that they are indeed closely related and indicative of clinical distress. Future studies could further validate these measures in the Pakistani population by evaluating psychological distress *via* another measurement tool. This could provide further evidence for the validity of these measurement tools in this context.

Most research into psychological illness and distress indicates that women report higher rates of mental illness. Indeed, this is the case of the recent survey of the levels of depression in undergraduate students in Pakistan in response to coronavirus (10). The unusual finding in this study that men have higher rates of anxiety about and obsessions related to coronavirus can be possibly explained by several factors. In Pakistan, men are more likely to be required to enter society and interact outside of the home. This could increase anxiety and obsessional thoughts in two ways. First, the likelihood of infection through exposure is higher for those going out and interacting within the society. Second, the need to go outside the home brings reminders of the situation; it is not possible to “pretend” that everything is normal. Furthermore, importantly, research indicates that men are more likely to have severe symptoms and have a higher mortality rate than women (18).

## Strengths and Limitations

There are several important strengths and also limitations of the present study. Strengths include that the study was completed during the lockdown period, when the pandemic had first begun. This provided important insights into the early stages of the pandemic, when people were first adjusting to the news of COVID-19. Being able to measure the levels of anxiety and obsessions regarding COVID-19 during this period provided a rare opportunity to examine the initial impact of the pandemic. Moreover, the size of the sample provided enough power to test the study hypotheses with confidence.

Limitations include the sampling method and the sample characteristics. Convenience sampling, particularly snowball sampling, is not the ideal method of obtaining a sample; however, given the circumstances, it was important to abide by the rules of the country that did not allow for in-person data collection or random sampling. Moreover, this sample is more highly educated than the general population of Pakistan. Though this study provides an accurate picture of the average educated person in Lahore, it cannot be generalized to other areas of Pakistan, particularly regions with fewer resources and a less educated population on average, especially given the known influence of education levels on stress.

## Conclusion

The COVID-19 pandemic is a rapidly changing situation with impacts on all areas of life. Recent studies show that mental health has been particularly negatively impacted by both the direct effects of the virus, as well as the secondary effects of such measures as lockdowns and the resultant economic tolls on the population. However, in this sample, rates of mental illness related to COVID-19 were found to be much lower than expected. Furthermore, importantly, in an unusual result, men were found to have higher rates of both anxiety and obsessional thinking related to COVID-19. These findings are intriguing and could be due to multiple factors, including lack of understanding about COVID-19, resilience, and culture. Future research should seek to replicate and further understand the psychological impact of COVID-19 on the general population of this developing nation as the pandemic continues to spread. Cultural factors as well as understanding of the situation and the impact of resilience should be considered.

## Data Availability Statement

The raw data supporting the conclusions of this article will be made available by the authors, without undue reservation.

## Ethics Statement

The studies involving human participants were reviewed and approved by Punjab Institute of Mental Health. The patients/participants provided their written informed consent to participate in this study.

## Author Contributions

SM conceived the idea of the study and developed the study protocol. All authors contributed to the data collection and analyses. All authors contributed to the writing and review of the manuscript for publication.

## Conflict of Interest

The authors declare that the research was conducted in the absence of any commercial or financial relationships that could be construed as a potential conflict of interest.
